# Antimicrobial Resistance in Papua New Guinea: A Narrative Scoping Review

**DOI:** 10.3390/antibiotics12121679

**Published:** 2023-11-29

**Authors:** Brady Page, Simeon Adiunegiya

**Affiliations:** 1 Department of Infectious Diseases and Global Public Health, University of California, San Diego (UCSD), La Jolla, CA 92103, USA; 2 Scripps Research Institute, La Jolla, CA 92037, USA; 3 School of Public Health, University of Washington, Seattle, WA 98195, USA

**Keywords:** antimicrobial resistance, Papua New Guinea, antibiotics, Oceania, Melanesia

## Abstract

Antimicrobial-resistant bacterial infections are a known threat to the public health of low-income countries and are undercharacterized in Papua New Guinea. A scoping literature review of scientific peer-reviewed publications on antimicrobial resistance in Papua New Guinea was conducted, and their results were summarized. Many of the available data on resistant bacteria in Papua New Guinea have come from Port Moresby and Goroka and have been focused on *Staphylococcus aureus*, as well as important pediatric pathogens such as *Streptococcus pneumoniae* and *Haemophilus influenzae*. Progressive resistance to the commonly used antibiotics penicillin and chloramphenicol among most clinically important bacterial pathogens has prompted healthcare workers to adopt expensive broad-spectrum antibiotics. There is already evidence of resistance to newly adopted antibiotics among several Gram-negative organisms. Drivers of antimicrobial resistance in Papua New Guinea include a high burden of infectious diseases, inappropriate antibiotic prescription practices, poor regulation of antibiotics, incomplete adherence, substandard drug quality, and overcrowding of healthcare facilities. There is a lack of information on antimicrobial resistance among priority pathogens and from several important regions of Papua New Guinea.

## 1. Introduction

**Figure 1:**
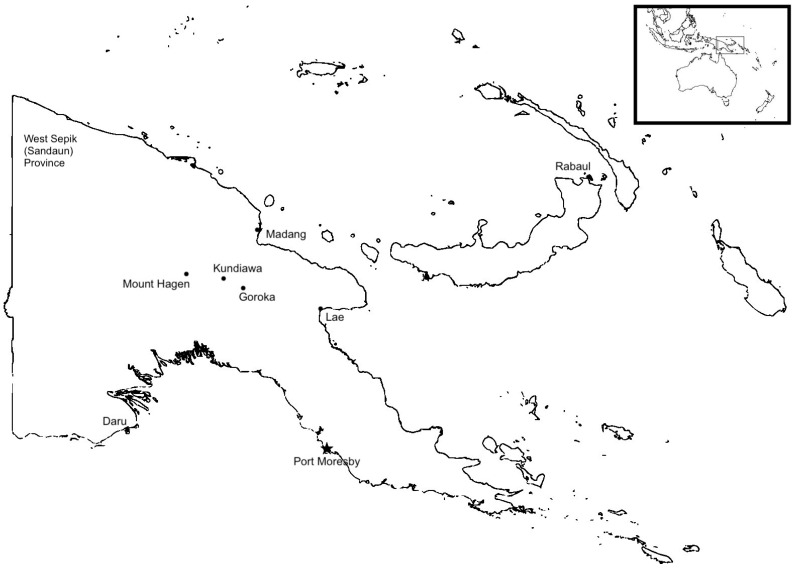
Map of Papua New Guinea and selected cities.

Papua New Guinea (PNG) is a Melanesian country in Oceania with a population of over 7 million that is composed of the eastern half of the island of New Guinea as well as hundreds of smaller islands scattered throughout the Solomon and Bismarck Seas [1]. The country is predominantly mountainous, with a tropical rainforest climate and densely populated central highlands. As a consequence of its dramatic geography, human communities in PNG have historically remained relatively isolated from one another, making it among the most culturally and biologically diverse regions on Earth (Figure 1).

As of 2020, the population of PNG is primarily rural, with only around 13% of Papuans living in urban settings and the remainder residing in villages—often in extremely remote locations. PNG is classified as a lower–middle-income country by the World Bank [2]. Its human development index is 155th in the world, and the life expectancy at birth is 67.8 years [3]. Infections are widespread in PNG—endemic and emerging infectious diseases are implicated in half of childhood deaths and over 40% of all mortality in the country [4,5].

The modern antibiotic era began in 1928 with the discovery of penicillin by Alexander Fleming [6]. Since then, antibiotics have transformed the medical field and saved countless lives; however, by the 1950s, penicillin resistance had already become a serious global problem [7]. Thus began the ongoing *contradanse* of new antibiotic development in the face of intensifying resistance.

The recognition and characterization of antimicrobial resistance (AMR) in high-income countries is well established, and data have emerged showing that the problem is more prevalent and, indeed, of higher magnitude in low-income countries, heralding a true global public health emergency [8,9]. Given the pervasiveness of infectious diseases and antibiotic use in PNG—coupled with the relative paucity of health infrastructure and resource capacity—it is worth examining the history and current state of AMR in the country.

To achieve these ends, this comprehensive scoping review will summarize knowledge and epidemiological trends with respect to AMR in PNG (as well as the former Australian-administered territory of Papua and New Guinea, prior to 1975) that have been disclosed in peer-reviewed surveys, case series, case reports, theses, or conference communications. Prevalence and sample size data pertaining to resistance among bacterial species or genera against individual antibiotics or antibiotic classes are reported, when available. There will then be a discussion of factors that contribute to AMR in PNG. This review will not address resistance in *Mycobacterium tuberculosis*, *Plasmodium* spp., or HIV, as these topics have been reviewed elsewhere [8,9,10].

## 2. Methods

**Figure 2:**
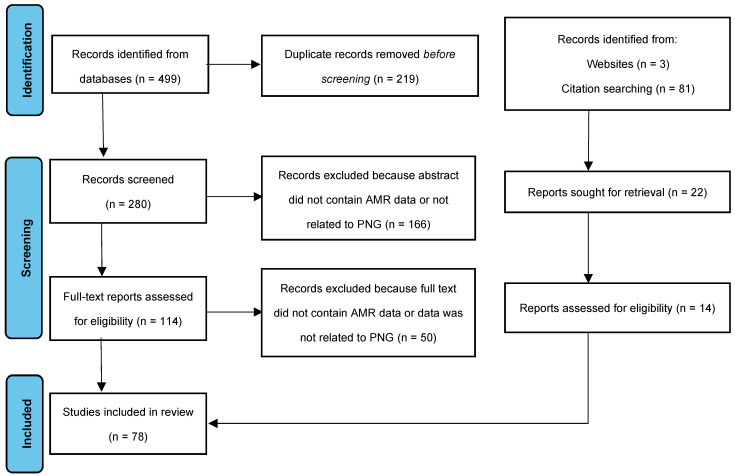
PRISMA chart of article identification and screening [11].

A literature review of scientific peer-reviewed publications was conducted following the Preferred Reporting Items for Systematic Reviews and Meta-Analyses Extension for Scoping Reviews (PRISMA-ScR) on the electronic databases PubMed and Web of Science. The search included combinations of the following keywords present in the title and/or abstract: “Papua”, “New Guinea”, “AMR”, and “antimicrobial resistance”. The format of publications could be prospective or retrospective studies, case reports, or case series, and they were evaluated to ensure that the contents represented commentary on antimicrobial susceptibility in what today constitutes the nation of Papua New Guinea. Antimicrobial susceptibility data were then extracted, and the AMR rates were calculated manually when not included in the publications (Figure 2).

### 2.1. Antimicrobial Resistance among Gram-Positive Organisms

#### 2.1.1. *Staphylococcus aureus*

**Figure 3:**
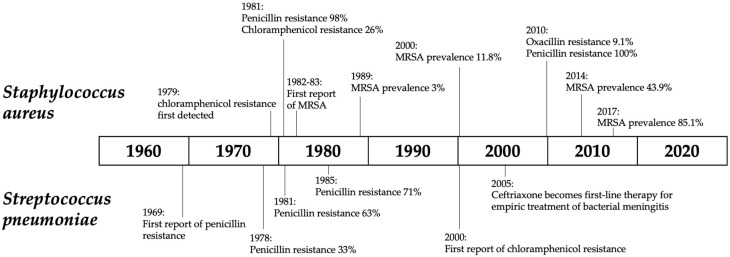
Timeline of antimicrobial resistance events for *Staphylococcus aureus* and *Streptococcus pneumoniae* in Papua New Guinea [9,10,12,14,15,16,17,18,19,20,21,22,23,24].

*S. aureus* is an important cause of a multitude of both community-acquired and nosocomial infections worldwide [12]. Between 1971 and 1981—a period during which penicillin was the first-line therapy for staphylococcal pyomyositis, acute osteomyelitis, pneumonia, and bacteremia in PNG—over 90% of *S. aureus* isolated from pus specimens at Port Moresby General Hospital was already penicillin-resistant (Supplementary Materials Table S1; Figure 3) [13]. 

During the last 3 years of this period, penicillin resistance had risen to 98%; however, no methicillin or gentamicin resistance was detected. A penicillin-resistant *S. aureus* that was sensitive to cloxacillin and gentamicin was cultured in 1978 from the pus and sputum of a 33-year-old man in Rabaul, East New Britain with gluteal pyomyositis and hematogenously seeded pneumonia [25].

In 1979, the first incidence of chloramphenicol-resistant *S. aureus* in PNG was identified in Port Moresby, and from 1979 to 1981 chloramphenicol resistance was detected in 26% of isolates [13].

The first methicillin-resistant *S. aureus* (MRSA) reported in PNG was isolated from the infected skin lesion of a child in Goroka at some point between 1982 and 1983, and it was thought to be community-acquired [15]. During the same period, MRSA was detected in a total of 0.75% (3/399) of *S. aureus* cultured from skin swabs from unrelated children in Goroka with infected skin lesions. These 3 MRSA isolates were also resistant to chloramphenicol, tetracycline, and erythromycin, while 98.2% (392/399) of all *S. aureus* isolates produced β-lactamase and were resistant to penicillin.

Also in Goroka, a survey of 73 *S. aureus* isolates from blood, CSF, urine, skin lesions, stool, joint aspirates, and lung tissue from patients of all ages between 1982 and 1986 found 97% (71/73) penicillin resistance, 7% (5/73) chloramphenicol resistance, 3% (2/66) tetracycline resistance, 1% (1/73) cotrimoxazole resistance, 1% (1/73) erythromycin resistance, and no resistance to gentamicin [16]. A survey of the bacteriology of children’s untreated skin sores in Goroka, Eastern Highlands in 1984 found that 92% (23/25) of *S. aureus* isolates were penicillin-resistant [26]. MRSA was cultured from one blood sample and one sputum sample, representing 3% (2/73) of all *S. aureus* isolates and the first report of MRSA in a hospitalized patient in PNG. MRSA was again isolated from the lung tissue of a 9-month-old with community-acquired pneumonia in Goroka in 1989 [27].

By the year 2000 there has been 3 deaths among children in Goroka from community-acquired MRSA infections that were also resistant to chloramphenicol, as well as reports of methicillin resistance in 11.8% (2/17) of *S. aureus* isolated from cases of fatal pediatric infection [14,28]. Oxacillin resistance was detected in 75% (3/4) of *S. aureus* isolated from the blood of surgical patients in Madang, Morobe Province from 2008 to 2009 [29]. All 3 isolates were susceptible to chloramphenicol and considered to have been community-acquired.

In 2010, a cross-sectional survey of *S. aureus* nasal colonization in Madang found oxacillin resistance in 9.1% (4/44) of isolates [17]. All of the isolates were penicillin-resistant (*n* = 44), 2.3% (1/44) were erythromycin-resistant, 2.3% (1/44) were trimethoprim–sulfamethoxazole-resistant, 4.6% (2/44) were tetracycline-resistant, and none were resistant to either rifampicin or clindamycin (*n* = 44).

In a 2013 report from Kundiawa, Simbu Province, more than 90% of *Staphylococcus* isolates from patients with osteomyelitis were resistant to flucloxacillin and chloramphenicol but retained universal susceptibility to ciprofloxacin [30].

By 2014, national data reported that 43.9% (72/164) of *S. aureus* cultured from blood, urine, and wounds was MRSA [18]. A prospective analysis of *S. aureus* isolates from the blood, joints, bone, or surrounding soft tissues of children from the community presenting with osteomyelitis in Kundiawa from 2012 to 2017 found that 85.1% (40/47) were methicillin-resistant, 89.4% (42/47) were oxacillin-resistant, 91.5% (43/47) were penicillin-resistant, 93.6% (44/47) were ampicillin-resistant, and 80.9% (38/47) were ceftriaxone-resistant [19]. There was also 8.5% (4/47) gentamicin resistance, 6.4% (3/47) erythromycin resistance, 6.4% (3/47) tetracycline resistance, 6.4% (3/47) clindamycin resistance, and 4.3% (2/47) cotrimoxazole resistance. 

#### 2.1.2. *Streptococcus pneumoniae*

*S. pneumoniae* is a highly invasive encapsulated bacterial pathogen and the most common cause of pneumonia, meningitis, bloodstream infections, and middle-ear infections in children [31]. The first known penicillin-resistant *S. pneumoniae* was detected in 1967 in the sputum of a patient with hypogammaglobulinemia and bronchiectasis in Sydney, Australia [20]. In April of 1969, the first penicillin-resistant *S. pneumoniae* in PNG was isolated from a throat swab collected from a healthy 3-year-old boy in Anguganak, West Sepik who had been given penicillin the previous year for pneumonia [32]. As part of a regional survey of Anguganak conducted during the same year, penicillin-resistant *S. pneumoniae* isolates from 15 more individuals that also demonstrated decreased susceptibility to cephaloridine, cephalothin, and methicillin while maintaining susceptibility to ampicillin were identified [33]. Ultimately, surveys conducted from 1968 to 1970 in West Sepik found that 12% (*n* = 530) of *S. pneumoniae* isolates were resistant to penicillin, with MICs (minimum inhibitory concentrations) that could be overcome in cases of pneumococcal pneumonia but posed a serious problem when attempting to achieve the therapeutic levels required to treat pneumococcal meningitis [34].

From 1971 to 1974, 14% (42/292) of *S. pneumoniae* isolates evaluated in PNG were penicillin-insensitive [32]. By 1978, 33% (19/57) of *S. pneumoniae* isolates from 23 children and 34 adults in Port Moresby with bacteremic pneumonia, meningitis, or bacteremia were resistant to penicillin [21]. No resistance to chloramphenicol, erythromycin, or tetracycline was detected at that time. By 1981, a prospective collection of lung aspirates and blood from children admitted to Goroka Hospital with pneumonia between 1978 and 1981 found 63% (15/24) of *S. pneumoniae* isolates to be penicillin-resistant [21]. In a subsequent survey taking place between 1980 and 1984 of CSF from children under 10 years old with purulent meningitis in Goroka Hospital, penicillin resistance in *S. pneumoniae* was 22% (15/67), and there was no resistance to chloramphenicol [35]. *S. pneumoniae* isolated from infected skin lesions of children in Goroka between 1982 and 1983 found 60% (3/5) penicillin resistance and preserved universal susceptibility to chloramphenicol [15].

In a large cohort of samples collected between 1980 and 1985 from the blood, CSF, joint aspirates, stool, lung tissue, urine, and skin lesions of patients of all ages in Goroka (with a small number of samples from Port Moresby), intermediate resistance to penicillin was detected in 56% (1701/3018) of *S. pneumoniae* isolates [16]. The prevalence of penicillin resistance was found to have increased throughout the duration of the survey, from 38% in 1980 to 71% in 1985. Among carriage and invasive isolates of *S. pneumoniae*, penicillin resistance was detected in 60% and 27% of isolates, respectively. Of 1047 isolates analyzed, all remained susceptible to chloramphenicol. From 1983 to 1984, 46% (13/28) of *S. pneumoniae* isolates from the blood of children with lower respiratory infections in Goroka were relatively penicillin-resistant, with MICs greater than 0.05 μg/mL, but remained sensitive to chloramphenicol [36].

Isolates of *S. pneumoniae* cultured from blood and lung aspirates collected from children with acute respiratory infections in Eastern Highlands between 1978 and 1987 demonstrated 52% (38/73) penicillin resistance [37]. Among carriage isolates from children between 1980 and 1982, there was 63% (602/956) resistance to penicillin, which had risen to nearly 75% by 1987 [38,39]. Analysis of a further 655 invasive isolates from lung aspirates or blood in the same region between 1985 and 1987 found no resistance to chloramphenicol, erythromycin, tetracycline, or cotrimoxazole [37]. 

In a survey carried out between 1980 and 1987 to compare the epidemiology and resistance patterns of carriage and invasive *S. pneumoniae* in Eastern Highlands, insensitivity to penicillin was detected in 67% (605/898) of isolates [40]. There was no significant change in the prevalence of penicillin insensitivity between the periods from 1980 to 1984 and from 1985 to 1987. Between 1989 and 1992, a prospective analysis of blood and CSF from children in Goroka with suspected bacterial meningitis found 22% (10/46) oxacillin resistance and 74% (45/61) cotrimoxazole resistance, as well as insensitivity to penicillin and trimethoprim in 23% (7/31) and 65% (20/32) of isolates, respectively [41]. In the same cohort, there was no resistance in *S. pneumoniae* to either chloramphenicol or erythromycin. Between 1998 and 2000, there was a single report in Eastern Highlands of a pediatric death from infection by penicillin-resistant *S. pneumoniae* that remained susceptible to chloramphenicol [14]. Between 1996 and 2000, the median MIC of chloramphenicol for *S. pneumoniae* was 3 μg/mL, and by 2000 chloramphenicol resistance was finally identified in 2 isolates of *S. pneumoniae* from children with bacterial meningitis in Goroka [22,23].

Between 1996 and 2005, prospective antimicrobial sensitivity testing of *S. pneumoniae* cultured from the CSF of children with meningitis in Goroka identified penicillin resistance in 21.5% (38/177) of isolates as well as 2.3% (4/176) chloramphenicol resistance, 4.2% (4/96) tetracycline resistance, and 4% (7/176) cotrimoxazole resistance [42]. Over the course of the survey, penicillin resistance decreased from 25% (29/116) to 14.8% (9/61), chloramphenicol resistance increased from 0.9% (1/115) to 4.9% (3/61), tetracycline resistance increased from 2.9% (2/69) to 7.4% (2/27), and cotrimoxazole resistance increased from 7.8% (9/116) to 10% (6/60).

In 2005, based on elucidated resistance patterns in *S. pneumoniae* and *H. influenzae*, the antibiotic of choice for the empiric management of bacterial meningitis in PNG was officially changed from chloramphenicol to ceftriaxone [24]. Although frank resistance to ceftriaxone had never been identified in PNG, 0.8% (1/124) of *S. pneumoniae* isolates from the CSF of children in Goroka between 1996 and 2005 were already intermediately resistant [42].

From 2006 to 2009, when the breakpoint for chloramphenicol resistance in *S. pneumoniae* was 16 μg/mL, among isolates from the blood and CSF of children with bacterial meningitis in Madang the median MIC of chloramphenicol was 3 μg/mL, and 42.8% (6/14) had an MIC of ≥4 μg/mL [23]. The likelihood of achieving a therapeutic AUC_0–24_/MIC with chloramphenicol against *S. pneumoniae* with an MIC of 4 was estimated to be 51–70%. This same survey detected 13.3% (2/15) penicillin resistance, 17.7% (3/17) trimethoprim–sulfamethoxazole resistance, 41% (7/17) trimethoprim–sulfamethoxazole insensitivity, 6.3% (1/16) tetracycline resistance, 25% (4/16) tetracycline insensitivity, and no resistance to ceftriaxone (*n* = 15).

In 2009, a retrospective analysis of invasive *S. pneumoniae* from patients with bacterial meningitis in Port Moresby identified chloramphenicol resistance in 8% (3/38) of isolates as well as 93.9% (31/33) gentamicin resistance, 16.7% (1/6) cotrimoxazole resistance, 7% (3/40) penicillin resistance, 4% (3/28) tetracycline resistance, and no resistance to ampicillin (*n* = 13), amoxicillin (*n* = 3), ceftriaxone (*n* = 6), erythromycin (*n* = 40), or cefaclor (*n* = 28) [43].

#### 2.1.3. *Streptococcus pyogenes* (Group A *Streptococcus*)

*S. pyogenes* is a common colonizer of the pharynx and skin and can cause pharyngitis, skin infections, and toxic shock syndrome [44]. A 1983 survey of skin swabs from children in Goroka with infected skin lesions found that all 337 isolates of *S. pyogenes* were penicillin-sensitive [15]. A prospective analysis of several body tissues in Goroka between 1984 and 1986 found no evidence of resistance to penicillin, chloramphenicol, or tetracycline [16].

#### 2.1.4. *Streptococcus agalactiae* (Group B *Streptococcus*)

Traditionally considered to be a neonatal pathogen, *S. agalactiae* is also associated with invasive disease in other high-risk groups [45]. Skin swabs collected as part of a prospective survey of children in Goroka with infected skin lesions from 1982 to 1983 found that 100% (*n* = 2) of *S. agalactiae* isolates were susceptible to penicillin [15]. Between 1998 and 2000, there was a report of a single pediatric death from pneumonia caused by multidrug-resistant *S. agalactiae* [14].

#### 2.1.5. Other β-Hemolytic *Streptococcus* spp.

Other β-hemolytic streptococci are colonizers of animals and humans and are associated with invasive infection in rare instances [46]. Analysis of isolates from blood, CSF, urine, skin lesions, stool, and lung tissue from Eastern Highlands between 1984 and 1986 found that 5% (1/22) of β-hemolytic streptococci were chloramphenicol-resistant, 15% (3/20) were tetracycline-resistant, and none (*n* = 22) were resistant to penicillin [16]. Specifically, there was one Group C *Streptococcus* isolated from a leprous foot ulcer that was the first instance of chloramphenicol resistance in this group in PNG, as well as 3 isolates that were tetracycline-resistant.

#### 2.1.6. *Corynebacterium* spp.

The corynebacteria are generally innocuous constituents of the human microflora that are occasionally implicated in skin and soft tissue, respiratory, genitourinary tract, or implanted device infections [47]. Most notable among them is *C. diphtheriae*, which causes diphtheria. In a survey of infected skin lesions collected between 1984 and 1986 in Eastern Highlands, no evidence of resistance to penicillin, chloramphenicol, erythromycin, or tetracycline was detected among 8 isolates of *C. diphtheriae* and *C. haemolyticum* (now *Arcanobacterium haemolyticum*) [16].

### 2.2. Antimicrobial Resistance among Gram-Negative Organisms

#### 2.2.1. *Haemophilus influenzae*

Prior to the introduction of a vaccine, *H. influenzae* type b was a common cause of invasive bacterial infection and bacterial meningitis in children [48]. Penicillin resistance in *H. influenzae* in PNG was first detected in 1981 in isolates from one CSF sample and one nasopharyngeal aspirate from 2 infants in Goroka as part of larger prospective analyses of children with meningitis or pneumonia [49,50]. Both isolates produced β-lactamase and were susceptible to chloramphenicol. During the same survey, the overall rate of penicillin-resistant *H. influenzae* was found to be only 0.7% (2/293). A survey of CSF from children with purulent meningitis in Goroka conducted between 1980 and 1984 found that only 1.8% (1/56) of *H. influenzae* isolates (83% of which were type b) were insensitive to penicillin and all isolates were susceptible to both chloramphenicol and ampicillin (Supplementary Materials Table S2) [35].

Among *H. influenzae* isolates from a number of bodily fluids and tissue types between 1981 and 1986 in Eastern Highlands, only 0.3% (4/1516) were resistant to penicillin and ampicillin [16]. The same survey found 100% susceptibility to chloramphenicol in 839 isolates analyzed from 1983 to 1986. 

Between 1983 and 1984, all isolates of *H. influenzae* from the blood of 30 children with lower respiratory infections in the same region were susceptible to ampicillin and chloramphenicol [36]. From 1989 to 1992, 13% (3/24) of *H. influenzae* type b isolates from the blood or CSF of children in Goroka with suspected bacterial meningitis were ampicillin-resistant, although none of the isolates produced β-lactamase (*n* = 32) [41]. In the same cohort, 28% (7/25) of isolates were cotrimoxazole-resistant, 44% (4/9) were trimethoprim-resistant, and there was still no resistance to either chloramphenicol or tetracycline.

Despite reliable efficacy throughout most of the twentieth century, in 1998 chloramphenicol resistance was detected in 25% of invasive *H. influenzae* type b isolates from children with bacterial meningitis in Lae, Goroka, and Port Moresby [51]. By 2000, chloramphenicol resistance in the same cohort was 21% [39]. Later that same year, resistance to chloramphenicol was similarly detected in 22.7% of *H. influenzae* isolates in Goroka, including 36.4% (4/11) of cases of fatal pediatric meningitis in the region [4,20].

During a survey conducted from 1996 to 2005, *H. influenzae* isolates from the CSF of children in Goroka with meningitis were 31.5% (51/162) chloramphenicol-resistant, 28.4% (46/162) ampicillin-resistant, and 34% (55/162) cotrimoxazole resistant, with 28% (38/165) of isolates demonstrating resistance to all three antibiotics [42]. Over the survey period, chloramphenicol resistance increased significantly from 26% (27/104) to 41.4% (24/58), ampicillin resistance increased significantly from 26% (27/104) to 46.6% (27/58), and cotrimoxazole resistance increased from 33.7% (35/104) to 48.3% (28/58). From this same cohort, 5% (4/80) of isolates were found to be non-susceptible to ceftriaxone. The observation of increasing chloramphenicol resistance in *H. influenzae* prompted the adoption of ceftriaxone for the empiric management of meningitis in PNG in 2005, with subsequent improvements in mortality [22,52].

From 2006 to 2009, 100% (*n* = 14) of *H. influenzae* isolates from the blood or CSF of children with bacterial meningitis in Madang were resistant to chloramphenicol [23]. Additionally, 93.3% (14/15) were penicillin-resistant, 100% (*n* = 15) were resistant to trimethoprim–sulfamethoxazole, 93.3% (14/15) were tetracycline-resistant, and there was no resistance to ceftriaxone (*n* = 10).

#### 2.2.2. *Escherichia coli*

*E. coli* is an environmental bacterium that commensally composes part of the normal mammalian gut microflora but can become pathogenic, most frequently implicated in genitourinary infections and gastroenteritis as well as pneumonia and meningitis [53]. Some strains are toxigenic, such as the enterohemorrhagic *E. coli* responsible for causing hemolytic uremic syndrome. Penicillin- and methicillin-resistant *E. coli* that was susceptible to chloramphenicol and gentamicin was isolated from the sputum of a man with *S. aureus* pneumonia in East New Britain in 1978 [25]. During a prospective survey of blood, urine, stool, CSF, lung tissue, and joint aspirates in Eastern Highlands from 1984 to 1986, ampicillin resistance was present in 46% (17/37) of isolates while 32% (12/27) were resistant to chloramphenicol, 11% (3/27) were resistant to tetracycline, 8% (3/37) were resistant to cotrimoxazole, 8% (1/13) were resistant to kanamycin, and 3% (1/37) were resistant to gentamicin [16]. *E. coli* isolates from children in Goroka with sepsis in 1997 and 1998 were 88% (7/8) chloramphenicol-resistant and 38% (3/8) gentamicin-resistant [54].

National data in 2012 reported that *E. coli* cultured from a variety of tissues demonstrated 24.1% (42/174) resistance to third-generation cephalosporins and 13.3% (70/526) resistance to fluoroquinolones [18].

#### 2.2.3. *Klebsiella* spp.

The *Klebsiella* are opportunistic pathogens implicated most frequently in nosocomial infections of the genitourinary tract, bloodstream, and respiratory system [55]. In 1978, a penicillin- and methicillin-resistant *Klebsiella* that remained susceptible to chloramphenicol and gentamicin was isolated from the sputum of a man with *S. aureus* pneumonia in Rabaul [25]. Among *Klebsiella* and *Enterobacter* isolates from blood, stool, urine, and lung tissue in Goroka between 1984 and 1986, 95% (21/22) were resistant to ampicillin while 45% (10/22) were resistant to chloramphenicol, 36% (4/11) were resistant to tetracycline, 32% (7/22) were resistant to cotrimoxazole, 6% (1/17) were kanamycin-resistant, and 5% (1/22) were gentamicin-resistant [16].

In 1992, there were 3 isolates of multidrug-resistant *Klebsiella* identified at Port Moresby General Hospital, including a single hospital-acquired *K. oxytoca* cultured from the blood of a 23-year-old man with pneumococcal pneumonia that was tetracycline-susceptible but resistant to penicillin, carbenicillin, ampicillin, chloramphenicol, gentamicin, cotrimoxazole, and streptomycin [56]. At that time, there was 54% (15/28) resistance to chloramphenicol, 61% (17/28) resistance to cotrimoxazole, and 29% (8/28) resistance to tetracycline in *K. pneumoniae* and *K. oxytoca* isolated from blood collected at the same hospital. 

*Klebsiella* collected in 1997 and 1998 from various sites of infection among children with sepsis in Goroka showed 100% (*n* = 14) resistance to chloramphenicol and 76% (11/14) resistance to gentamicin [54].

From October 2007 to October 2008, there was an outbreak of nosocomial sepsis due to multidrug-resistant *K. pneumoniae* in the special care nursery at Port Moresby General Hospital. During the first 3 months of the outbreak, 20% (4/20) of *K. pneumoniae* isolates cultured from blood were found to be resistant to cephalosporins [57]. Over the ensuing 10 months, cephalosporin resistance increased to 73% (27/37), with 19% (6/31) of isolates demonstrating resistance to all available antibiotics. 

From 2008 to 2009 in Madang, *K. pneumoniae* that was isolated from bacteremic surgical patients was 100% (*n* = 2) resistant to ampicillin, chloramphenicol, cotrimoxazole, and tetracycline, 50% (1/2) resistant to gentamicin, and completely susceptible to ciprofloxacin (*n* = 2) [29]. By 2012, 63.5% (160/252) of *Klebsiella* isolates from blood, urine, stool, wounds, and pus from around PNG were resistant to third-generation cephalosporins [18].

#### 2.2.4. *Enterobacter* spp.

*Enterobacter* spp. are responsible for nosocomial infections of the genitourinary tract, bloodstream, and respiratory system and are naturally resistant to penicillins, first- and second-generation cephalosporins, and amoxicillin–clavulanic acid [58]. Resistance in *Enterobacter* and *Klebsiella* spp. to ampicillin, chloramphenicol, tetracycline, cotrimoxazole, and aminoglycosides was detected in Eastern Highlands from 1984 to 1986, as above. From 1997 to 1998, again in Eastern Highlands, chloramphenicol resistance was detected in 100% (7/7) and gentamicin resistance in 57% (4/7) of isolates from children with severe sepsis [54].

#### 2.2.5. *Proteus* spp.

*Proteus* spp. are urease-producing bacilli that are uncommonly implicated in catheter-associated urinary-tract and wound infections that can invade to cause nosocomial bacteremia [59]. Resistance to ampicillin was present in 45% (5/11) of *Proteus* spp. and *Providencia* spp. isolated from urine, blood, stool, CSF, and skin lesions in Goroka between 1984 and 1986 [16]. This same survey detected resistance to chloramphenicol in 82% (9/11), resistance to tetracycline in 100% (*n* = 10), resistance to cotrimoxazole in 55% (6/11), resistance to gentamicin in 18% (2/11), and resistance to kanamycin in 33% (3/9) of isolates. Chloramphenicol resistance was present in 100% (*n* = 3) and gentamicin resistance in 33% (1/3) of *P. mirabilis* isolated from children in Goroka with sepsis between 1997 and 1998 [54].

#### 2.2.6. *Providencia* spp.

*Providencia* spp. are environmental organisms that are an uncommon cause of catheter-associated urinary-tract infections and a rare cause of nosocomial bacteremia [60]. Between 1984 and 1986, a prospective analysis of isolates from urine, blood, stool, and skin lesions in Goroka found resistance to ampicillin, chloramphenicol, tetracycline, cotrimoxazole, and aminoglycosides in *Providencia* spp. and *Proteus* spp., as above. A single isolate of *Providencia* from a child in Goroka with sepsis between 1997 and 1998 was susceptible to both gentamicin and chloramphenicol [54].

#### 2.2.7. *Morganella morganii*

*M. morganii* is commensal in the gastrointestinal flora of many animals and is rarely implicated in nosocomial urinary-tract and wound infections [61]. There was no evidence of gentamicin resistance (*n* = 2) or chloramphenicol resistance (*n* = 2) in isolates of *M. morganii* cultured from children with sepsis in 1997 and 1998 in Goroka [54].

#### 2.2.8. *Pseudomonas* spp.

The *Pseudomonas* spp., particularly *P. aeruginosa*, are environmental organisms with high levels of AMR that can cause severe opportunistic infections of many different organ systems, especially in immunocompromised hosts [62]. In a prospective analysis of blood, skin lesions, urine, CSF, joint fluid, and lung tissue from Goroka between 1984 and 1986, isolates of *Pseudomonas* spp. demonstrated 83% (10/12) resistance to chloramphenicol, 55% (6/11) resistance to ampicillin, 50% (4/8) resistance to tetracycline, 67% (8/12) resistance to cotrimoxazole, 30% (3/10) resistance to kanamycin, and no resistance (*n* = 12) to gentamicin [16]. Isolates of *P. aeruginosa* were 100% resistant to ampicillin, chloramphenicol, tetracycline, and cotrimoxazole. Isolates of *P. aeruginosa* from several sites of infection among children with severe sepsis in Goroka between 1997 and 1998 were 100% (*n* = 11) resistant to chloramphenicol and 82% (9/11) resistant to gentamicin [54].

A single *P. aeruginosa* cultured from the blood of a bacteremic surgical patient in Madang between 2008 and 2009 was resistant to ampicillin, tetracycline, cotrimoxazole, and chloramphenicol but remained susceptible to ciprofloxacin and gentamicin [29].

#### 2.2.9. *Acinetobacter* spp.

*Acinetobacter* spp. are multidrug-resistant environmental organisms and an important emerging cause of nosocomial urinary-tract, respiratory, bloodstream, and wound infections in critically ill patients [63]. In a case series of 5 community-acquired pneumonia cases in Port Moresby from 1986 to 1987, *A. calcoaceticus* was isolated from four percutaneous pulmonary aspirates and one blood culture [64]. Penicillin insensitivity was suggested, as the 2 patients who received only penicillin died and the 3 who received additional gentamicin survived their illnesses. 

A single isolate of *Acinetobacter* from a child in Goroka with sepsis between 1997 and 1998 was resistant to chloramphenicol but susceptible to gentamicin [54].

#### 2.2.10. *Burkholderia* spp.

*B. cepacia* is an emerging cause of multidrug-resistant pneumonia in cystic fibrosis patients, while *B. pseudomallei* causes a severe multiorgan infection known as melioidosis [65,66]. *B. cepacia* cultured from children with sepsis in Goroka from 1997 to 1998 was 100% resistant to gentamicin and 67% (2/3) resistant to chloramphenicol [54]. A retrospective analysis of clinical and environmental isolates of *B. pseudomallei* from Balimo, Western Province between 1995 and 2005 identified 48.7% (19/39) chloramphenicol resistance and universal susceptibility to tetracycline, amoxicillin–clavulanate, and meropenem (*n* = 39) [67].

#### 2.2.11. *Aeromonas* spp.

The *Aeromonas* spp. are environmental organisms of fresh or brackish water that are rarely implicated in intraabdominal, respiratory, gastrointestinal, genitourinary, bloodstream, and traumatic skin and soft-tissue infections—especially those associated with animal bites [68]. Among isolates of *A. hydrophila* cultured from stool and skin lesions in Eastern Highlands between 1984 and 1986, ampicillin resistance was 75% (6/8), chloramphenicol resistance was 13% (1/8), and there was no resistance to gentamicin (*n* = 8), cotrimoxazole (*n* = 8), or tetracycline (*n* = 5) [16]. A lone isolate of *Aeromonas* from a child with sepsis in Goroka between 1997 and 1998 was sensitive to gentamicin but resistant to chloramphenicol [54].

#### 2.2.12. *Citrobacter freundii*

*C. freundii* is an environmental and human intestinal commensal organism that is an uncommon cause of gastroenteritis as well as nosocomial urinary-tract infections, pneumonia, and bacteremia [69]. A prospective observational study over 16 months from 1997 to 1998 among children with sepsis in Goroka found that *C. freundii* isolates from multiple tissue types were 100% (3/3) gentamicin-resistant and 67% (2/3) chloramphenicol-resistant [54].

#### 2.2.13. *Alcaligenes* spp.

Bacteria in the genus *Alcaligenes* are multidrug-resistant environmental organisms that are rarely implicated in a multitude of opportunistic infections [70]. A single isolate of *Alcaligenes* from a child with sepsis in Goroka between 1997 and 1998 was susceptible to gentamicin and resistant to chloramphenicol [54]. A second *Alcaligenes* cultured from the blood of a bacteremic surgical patient in Madang between 2008 and 2009 was resistant to ampicillin and tetracycline, intermediately resistant to gentamicin and ciprofloxacin, and susceptible to chloramphenicol and cotrimoxazole [29].

#### 2.2.14. *Shigella* spp.

There are four major species of *Shigella*, which are highly virulent pathogens that carry a significant burden of gastrointestinal illness worldwide, especially in resource-limited settings [71]. Toxin-producing subtypes of *S. dysenteriae* can cause hemolytic uremic syndrome. A prospective analysis of 851 stool samples from patients with acute gastroenteritis in Port Moresby between 1962 and 1963 identified 75% (120/160) as *S*. *flexneri* and 15.6% (25/160) as *S*. *sonnei* [72]. Of the *Shigella* spp. isolates that were tested, 12.9% (9/70) were resistant to streptomycin and 8.6% (6/70) were resistant to oxytetracycline. Resistance to sulfonamides was found in 38.9% (14/36) of isolates, all of which were *S*. *flexneri*. 

An analysis of stool samples in Eastern Highlands from 1984 to 1986 detected ampicillin resistance in 86% (81/94) of *Shigella* isolates, while tetracycline and cotrimoxazole resistance was found in 91% (49/54) and 1% (1/94) of isolates, respectively [16]. Among the 94 isolates tested, there was no resistance to either kanamycin or gentamicin. Resistance to chloramphenicol was detected in 83% (78/94) of *Shigella* spp. isolates, all of which were *S. flexneri* serotypes 1 or 3. Among these *S. flexneri* isolates, 35% were resistant to 2 antibiotics, 48% were resistant to 3 antibiotics, and 1% were resistant to 4 antibiotics.

Between 2000 and 2009, the antibiotic susceptibilities of *Shigella* isolated from the stool of patients with severe diarrhea in Port Moresby—composed of 90.4% *S. flexneri*, 3.7% *S. boydii*, 2.9% *S. dysenteriae*, and 1.5% *S. sonnei*—were analyzed [73]. Among all combined *Shigella* spp. tested, 96% (94/98) were resistant to amoxicillin, 86% (65/76) were resistant to cotrimoxazole, 60% (68/114) were resistant to chloramphenicol, 27% (31/114) were intermediately resistant to chloramphenicol, and 15% (2/13) were resistant to nalidixic acid. No isolates were resistant to ciprofloxacin or cephalexin, but one isolate was intermediately resistant to ciprofloxacin and another was intermediately resistant to cephalexin.

Among 30 *S. flexneri*, 2 *S. dysenteriae*, and 15 non-typed *Shigella* spp. isolates from Eastern Highlands between 2010 and 2011, 91.5% (43/47) were ampicillin-resistant, 76.6% (36/47) were tetracycline-resistant, 70.2% (33/47) were resistant to trimethoprim–sulfamethoxazole, and 55.3% (26/47) were chloramphenicol-resistant [74]. There was no resistance to ceftriaxone or ciprofloxacin (*n* = 47). Resistance to 4 antibiotics was detected in 55.3% (26/47) of isolates, while resistance to 3 antibiotics was detected in 21.3% (10/47) of isolates. As of 2014, there was no resistance to fluoroquinolones (*n* = 53) among *Shigella* isolates from several sites in PNG [18].

A retrospective analysis in 2018 of archived stool from throughout Oceania—which included 60 samples from PNG—found that among isolates of *S. flexneri,* 77% (41/53) were ampicillin-resistant, 74% (39/53) were tetracycline-resistant, 60% (32/53) were chloramphenicol-resistant, and 49% (26/53) were trimethoprim–sulfamethoxazole-resistant [75]. Among isolates of *S. sonnei*, there was 56% (9/16) resistance to ampicillin, 19% (3/16) resistance to tetracycline, 75% (12/16) resistance to trimethoprim–sulfamethoxazole, and 6% (1/16) resistance to nalidixic acid. *S. dysenteriae* isolates were 33% (1/3) ampicillin-resistant, 33% (1/3) tetracycline-resistant, and 33% (1/3) trimethoprim–sulfamethoxazole-resistant. There was a significant increase in resistance to several antibiotics among *Shigella* spp. isolates when comparing isolates collected before and after 2010. Specifically, ampicillin resistance increased from 14% to 58%, chloramphenicol resistance increased from 10% to 35%, tetracycline resistance increased from 14% to 46%, and trimethoprim–sulfamethoxazole resistance increased from 8% to 48%. There was no resistance to ceftriaxone or ciprofloxacin identified.

#### 2.2.15. *Salmonella* spp.

*Salmonella* spp. are associated with acute gastroenteritis, usually caused by the consumption of contaminated water and food, or through contact with colonized animals [76]. Invasive disease—known as typhoid fever—is caused by *S. enterica* subsp. enterica serovar Typhi and can be life-threatening. From 1984 to 1986, a prospective analysis of blood and stool from Eastern Highlands demonstrated that 58% (22/38) of *Salmonella* spp. isolates were chloramphenicol-resistant, 53% (20/38) were ampicillin-resistant, 37% (14/38) were kanamycin-resistant, 6% (2/33) were tetracycline-resistant, 3% (1/38) were cotrimoxazole-resistant, and all (*n* = 38) were susceptible to gentamicin [16]. There was no resistance in *S. typhi*. Of the isolates tested, 11% were resistant to 2 antibiotics, 40% were resistant to 3 antibiotics, and 8% were resistant to 4 antibiotics. By the year 2000, *S. typhi* was broadly considered to be chloramphenicol-resistant in PNG [28].

A small number of *S. typhi* isolates from children and adults in Eastern Highlands in 2010 and 2011 found that 80% (4/5) were ampicillin-resistant, 60% (3/5) were tetracycline-resistant, 60% (3/5) were trimethoprim–sulfamethoxazole-resistant, and 40% (2/5) were chloramphenicol-resistant [74]. There was no resistance to ceftriaxone or ciprofloxacin (*n* = 5). In 2014, 33.3% (5/15) of non-typhoidal *Salmonella* isolates from around the country were resistant to fluoroquinolones [18].

#### 2.2.16. *Campylobacter* spp.

*Campylobacter* is an important cause of foodborne diarrheal illness in the highlands of PNG [77]. Among 22 *C. jejuni* and 33 *C. coli* isolates from stool in Eastern Highlands between 1984 and 1986, there was 24% (13/55) resistance to ampicillin, 100% (*n* = 55) resistance to cotrimoxazole, and no resistance to gentamicin, chloramphenicol, or tetracycline (*n* = 55) [16].

#### 2.2.17. *Vibrio cholerae*

Toxigenic *V. cholerae* is an outbreak-prone environmental organism of brackish water whose transmission via the fecal–oral route in areas of poor sanitation and poverty can elicit a disease characterized by devastating watery diarrhea and rapid dehydration, known as cholera [78]. An outbreak of *V. cholerae* serogroup O1, biotype El Tor, serotype Ogawa began in Morobe Province in 2009 and then spread to nearly half of the provinces of PNG by 2011. Among stool samples and rectal swabs collected during the outbreak between 2009 and 2011, 75.8% (229/302) of *V. cholerae* isolates were resistant to amoxicillin, and 17.2% (52/302) were intermediately resistant [79]. Chloramphenicol resistance was detected in 3.1% (8/255) of isolates, and 1.6% (4/244) were intermediately resistant. There was no resistance to norfloxacin (*n* = 296), but 0.7% (2/296) of isolates were intermediately resistant. Ciprofloxacin resistance was present in 1% (3/305) of isolates, and 3.2% (9/282) of isolates were resistant to cotrimoxazole. Similarly, 0.7% (2/305) of isolates were intermediately resistant to ciprofloxacin, and 1.4% (4/282) were intermediately resistant to cotrimoxazole. There was 0.3% (1/300) resistance to nalidixic acid. 

In 2009, the first year of the cholera outbreak, 27.8% (10/36) of isolates demonstrated at least intermediate resistance to tetracycline [79]. By 2010, this figure had risen to 50.5% (107/212), and it had decreased to 11.8% (6/51) by 2011. The overall rate of resistance to tetracycline during the outbreak was 9.7% (29/299). Erythromycin resistance was detected in 38.2% (97/254) of isolates, with the percentage of *V. cholerae* isolates that were at least intermediately resistant to erythromycin increasing from 92.1% (187/203) in 2010 to 96.1% (49/51) by 2011.

#### 2.2.18. *Neisseria meningitidis*

*N. meningitidis*—or meningococcus—is a strictly human pathogen that causes significant morbidity and mortality among non-immunized children and young adults through epidemic or sporadic meningitis with bacteremia [80]. A prospective survey from 1980 to 1984 in Goroka isolated *N. meningitidis* from 5.2% (8/155) of CSF samples from children with purulent meningitis [81]. A survey from 1984 to 1986, also in Goroka, found no evidence of resistance to penicillin or chloramphenicol in 5 isolates of *N. meningitidis* from CSF or blood [16]. In 2009, a small retrospective analysis of *N. meningitidis* isolates from patients with bacterial meningitis in Port Moresby identified 33% (1/3) resistance to ceftazidime, 33% (1/3) resistance to tetracycline, and no resistance to penicillin or ceftriaxone [43].

#### 2.2.19. *Neisseria gonorrhoeae*

*N. gonorrhoeae*, or gonococcus, is a common cause of sexually transmitted urethritis and cervicitis that can progress into pelvic inflammatory disease or disseminate to other organ systems [82]. Certain strains of *N. gonorrhoeae* have rapidly developed resistance to all antibiotic classes except for extended-spectrum cephalosporins, with some regions already grappling with ceftriaxone-resistant strains [83]. Penicillinase-producing *N. gonorrhoeae* represented 44% of all gonococcal isolates from sexually transmitted infection clinics in 5 towns across PNG in 1989 and 1990 [81]. Beginning in 1992, low but consistent levels of spectinomycin resistance began to be detected in the country [84]. In 1993, national data described penicillin resistance in 12.5% (5/40), tetracycline resistance in 7.5% (3/40), and spectinomycin resistance in 3.3% (1/30) of isolates with no evidence of fluoroquinolone resistance (*n* = 40) [85]. By 1994, penicillin resistance was 8.7% (19/218), tetracycline resistance was 4.1% (9/218), spectinomycin resistance was 1.8% (1/57), and resistance to fluoroquinolones was detected in 5% (11/218) of isolates. As of 1994, no resistance to third-generation cephalosporins had been detected in PNG [84].

By 2005, ceftriaxone resistance was detected at an extremely low level in Port Moresby, with only 0.7% of several hundred isolates demonstrating reduced susceptibility [86]. That same year, 1.2% ciprofloxacin resistance, 61.1% penicillin resistance, 49% tetracycline resistance, 0.7% spectinomycin resistance, and 5% nalidixic acid resistance were recognized. In 2006, ciprofloxacin resistance was 1.5%, penicillin resistance was 64.7%, tetracycline resistance was 17.7%, nalidixic acid resistance was 2.9%, and there was no evidence of resistance to either ceftriaxone or spectinomycin in Port Moresby.

A further 52 isolates collected from STI clinics in Port Moresby, Lae, Mount Hagen, and Goroka in 2004 and 2005 were all susceptible to amoxicillin–clavulanate, spectinomycin, erythromycin, azithromycin, and ceftriaxone [87]. However, 19% (10/52) of isolates were resistant to tetracycline, and 2% (1/52)—isolated in Lae—were resistant to ciprofloxacin. Penicillin resistance due to penicillinase was detected in 40% (21/52) of isolates. 

### 2.3. Drivers of Antimicrobial Resistance in Papua New Guinea

There has historically been a high burden of infectious diseases in PNG that warrant antibiotic treatment, as well as a pervasive presence of risk factors for infection including malnutrition, home birth, and prolonged hospitalization [28]. However, antibiotic misuse is a known cause of the development of AMR. Initially, penicillin was widely employed in PNG after World War II, with the establishment of the aid post system of primary healthcare [37]. It was used liberally, perhaps indiscriminately, at the village level for respiratory infections and for the eradication of yaws, even in remote areas [33]. In fact, data suggest that in the 10 years following 1961 the amount of penicillin used by the PNG Department of Health was equivalent to 10,670,000 5-day courses of the antibiotic [88].

Given the lack of robust microbiological laboratory capacity at many centers in PNG, practitioners sometimes resort to the empiric administration of broad or redundant antibiotics, as is the case with the management of genital discharge in PNG [89,90]. In healthcare facilities without the capacity to perform bacterial cultures or antimicrobial sensitivity testing, the only clue to AMR may be an increase in clinical failure in appropriately treated infections [22]. In the absence of routinely generated clinical microbiological data, deliberate research efforts have often been necessary in order to uncover the true state of resistance patterns in PNG [91].

Unofficial use of antibiotics has emerged as an important driver of AMR in PNG. Non-prescription dispensing of amoxicillin, chloramphenicol, and trimethoprim–sulfamethoxazole is common in the PNG highlands, where infection due to multidrug-resistant Gram-negative organisms has become a common cause of death [54]. In Popondetta, nearly all children with the common cold receive empiric antibiotics, with 30% of healthcare workers believing that antibiotics were indicated in this setting [92]. A significant number of patients in PNG have already received antibiotics by the time they present to a healthcare facility, indicating a need to regulate commercial pharmacies and provide education to health workers about appropriate antibiotic use [28,91].

Poor adherence to oral antibiotics can foster AMR [93]. The development of ampicillin resistance among Gram-negative organisms may be related to difficulties in adhering to newer dosing schedules that are more complicated compared to older approaches to the outpatient management of pneumonia [28]. Resistance to gentamicin amongst Gram-negative organisms has remained relatively low, probably due to the difficulty in the non-prescription dispensing and administration of intravenous medication. Throughout the twentieth century, third-generation cephalosporin use in PNG was extremely limited, minimizing opportunities for the development of resistance [94].

Even in the context of responsible antibiotic prescription practices and patient adherence, the circulation of poor-quality drugs in under-resourced regions has the potential to generate AMR [95]. Substandard or falsified drugs with inappropriate reductions in active pharmaceutical ingredients have been detected throughout the PNG supply chain and have been associated with inadequate manufacturing, quality control, and regulatory practices [96,97].

There is growing awareness amidst intensifying globalization that the emergence of resistant organisms in one part of the world can lead to an international problem [98]. Even if PNG manages to address the issues described above, growing interconnectedness with Asia and Australia—particularly through PNG’s productive extractive industries—raises the possibility that AMR could enter from abroad [99].

Other factors contributing to the spread of resistant organisms in PNG include overcrowding with low nurse-to-patient ratios and the absence of effective hygiene practices, both within hospitals and in the community [57].

## 3. Discussion and Conclusions

This is the first comprehensive review to specifically address the problem of AMR in PNG. Much of the effort on AMR in PNG has focused on *S. pneumoniae* and *H. influenzae*, as these agents were responsible for a high degree of mortality in childhood meningitis and pneumonia in the pre-vaccine age [35].

Historically, penicillin was the drug of choice for the treatment of infections caused by *S. pneumoniae* in PNG [100]. Resistance developed early and quickly, but susceptibility to chloramphenicol persisted throughout the twentieth century, prompting its adoption as the empiric treatment for childhood pneumonia and meningitis. Nonetheless, evolving resistance patterns among *S. pneumoniae* and *H. influenzae* dictated a broadening of the empiric treatment of bacterial meningitis in PNG to ceftriaxone in 2005. In 2014, the 13-valent pneumococcal conjugate vaccine (PCV13) was introduced into PNG’s national immunization program; however, coverage of the complete three-dose schedule in 2015 was estimated to be between 4% and 6.5% [42].

Chloramphenicol resistance among *H. influenzae* was absent for most of the twentieth century in PNG but became established quickly after it was initially detected in the late 1990s, generating concerns that expensive broad-spectrum antibiotics would soon be needed in order to empirically treat children with meningitis [22]. Fortunately, *H. influenzae* type b vaccination was included in PNG’s expanded program of immunization in 2008 [52]. By 2016, between 43% and 47.7% of children surveyed in Eastern Highlands had received three doses of the DTPw-HepB-Hib vaccine [91]. The integration of available vaccines—especially against *S. pneumoniae* and *H. influenzae* type b—into routine childhood immunization schedules has since led to reductions in childhood mortality [42].

Over time, the country has seen a marked increase in resistance to penicillin, methicillin, and oxacillin among *S. aureus*. There is already considerable circulation of MRSA in the community in PNG [19].

*Shigella* and *Salmonella* spp. appear to remain susceptible to ceftriaxone, but fluoroquinolone resistance has developed in *Salmonella* [16,18]. Ampicillin, chloramphenicol, and tetracycline are not good options for the treatment of infections associated with these organisms in PNG.

There is widespread, high-level resistance among *N. gonorrhoeae* to penicillin, azithromycin, and ciprofloxacin in the Western Pacific, as well as increased MICs of ceftriaxone and other extended-spectrum cephalosporins [84,101]. There is sporadic low-level resistance to fluoroquinolones and ceftriaxone among *N. gonorrhoeae* in PNG.

Resistant Gram-negative organisms—especially extended-spectrum β-lactamase (ESBL)-producing Enterobacterales—have been identified as a serious threat to human health in the twenty-first century [102]. There is a high degree of known resistance among Gram-negative organisms to a number of common antibiotics in PNG, including significant resistance to third-generation cephalosporins among the Enterobacterales [14,28]. It is possible that there is also substantial undetected AMR among Gram-negative organisms to newly available antibiotics such as ciprofloxacin, second- and third-generation cephalosporins, and other broad-spectrum parenteral antibiotics that have entered into empiric use in PNG. 

Broad-spectrum antibiotic resistance among Gram-negative organisms is concerning due to the high mortality rate associated with the progression of Gram-negative infections into sepsis [14]. Investigations of the prevalence of ESBL-producing organisms in PNG have been extremely limited, and the sample sizes in surveys of Gram-negative resistance have been small [29]. There is even more of a dearth of data from PNG on microbial susceptibility to carbapenems—which, along with other parenteral antibiotics such as vancomycin, daptomycin, lincomycin, linezolid, and ceftaroline, are not readily available in much of PNG [17,18]. It is likely that access to broader-spectrum antibiotics as a solution to growing AMR would only precipitate resistance to them [57].

There are no published data from PNG on AMR among *Enterococcus* spp., a genus of coliform bacteria associated with nosocomial infections and prone to plasmid-mediated resistance to vancomycin and beta-lactams [102].

The overwhelming majority of AMR data from PNG come from Port Moresby and Goroka, since these are the only centers where sufficient resources have traditionally existed to perform the required microbiological techniques [28]. As a result, little is known about patterns of resistance in other parts of the country. Ongoing sentinel surveillance for multidrug-resistant organisms should be continued in several carefully selected sites—including the Sepik, Daru, and outer insular regions, which have been largely excluded from AMR studies—that represent the extreme regional diversity within PNG [28,29].

Compared to the rest of the world, Oceania has reported more deaths associated with AMR than all regions except for southern Latin America, South Asia, and sub-Saharan Africa [103]. Among Pacific island countries, PNG boasts some of the best-characterized AMR and may harbor some of the highest rates of resistance [104].

As with any region, the increasing availability of newer and more powerful antibiotics in PNG will inevitably engender the development of broad and potentially novel AMR. With increasing connectivity to the world, it is easier than ever for resistant organisms or traits to spread to and from PNG [105,106]. Given these certitudes, further research should emphasize pathogens that have been identified as priorities by the WHO and for which there are currently scarce data from PNG, especially ESBL-producing and carbapenem-resistant Gram-negative organisms, cephalosporin- and fluoroquinolone-resistant *Neisseria gonorrhoeae*, and vancomycin-resistant *Enterococcus* [107].

## Data Availability

All data are available as outlined in the References.

## Supplementary Materials

The following supporting information can be downloaded at: https://www.mdpi.com/article/10.3390/antibiotics12121679/s1, Table S1: Antimicrobial resistance among Gram-positive organisms in Papua New Guinea; Table S2: Antimicrobial resistance among Gram-negative organisms in Papua New Guinea.
