# Supplementary material for: Antimicrobial Resistance in Papua New Guinea: A Narrative Scoping Review
Source: Antibiotics (Basel). 2023 Nov 29;12(12):1679. doi: 10.3390/antibiotics12121679 (PMC10741211; doi:10.3390/antibiotics12121679)
Supplement: Supplementary file 1 [file antibiotics-12-01679-s001.zip › antibiotics-2688619-supplementary.pdf]

**Table S1.** Antimicrobial resistance among Gram-positive organisms in Papua New Guinea.

| | | | % Resistance to Antibiotic (No. of Isolates Evaluated) | | | | | | | | | | | | | | |
|---|---|---|---|---|---|---|---|---|---|---|---|---|---|---|---|---|---|
| **Year** | **Location** | **Tissue** | **PCN** | **CLX** | **MET** | **OXA** | **AMP** | **CTX** | **GEN** | **CMP** | **TET** | **ERY** | **COT** | **TMP** | **CLN** | **CIP** | **RIF** |
| ***Staphylococcus aureus*** | | | | | | | | | | | | | | | | | |
| 1971–1981 [14] | Port Moresby | Pus | 90 | | 0 | | | | 0 | | | | | | | | |
| 1978 [15] | Rabaul | Pus, sputum | | 0 (1) | | | | | 0 (1) | | | | | | | | |
| 1979 [14] | Port Moresby | | | | | | | | | 100 (1) | | | | | | | |
| 1979–1981 [15] | Port Moresby | Pus | 98 | | 0 | | | | 0 | | | | | | | | |
| 1979–1981 [15] | Port Moresby | | | | | | | | | 26 | | | | | | | |
| 1982–1983 [16] | Goroka | Skin | | | | | 100 (1) | | | | | | | | | | |
| 1982–1983 [16] | Goroka | Skin | 98.2 (399) | | | | 0.75 (399) | | | | | | | | | | |
| 1982–1986 [17] | Goroka | Multiple † | 97 (73) | | 3 (73) | | | | 0 (73) | 7 (73) | 3 (66) | 1 (73) | 1 (73) | | | | |
| 1984 [18] | Goroka | Skin | 92 (25) | | | | | | | | | | | | | | |
| 1989 [19] | Goroka | Lung | | | 100 (1) | | | | | | | | | | | | |
| 2000 [20] | Goroka | | | | 11.8 (17) | | | | | | | | | | | | |
| 2008–2009 [21] | Madang | Blood | | | | 75 (4) | | | | 0 (3) | | | | | | | |
| 2010 [22] | Madang | Nasal swabs | 100 (44) | | | 9.1 (44) | | | | | 4.6 (44) | 2.3 (44) | | 2.3 (44) | 0 (44) | | 0 (44) |
| 2013 [23] | Kundiawa | | | 90 | | | | | | 90 | | | | | | 100 | |
| 2012–2017 [25] | Kundiawa | Blood, bone, joint aspirates, soft tissue | 91.5 (47) | | 85.1 (47) | 89.4 (47) | 93.6 (47) | 80.9 (47) | 8.5 (47) | | 6.4 (47) | 6.4 (47) | 4.3 (47) | | 6.4 (47) | | |
| 2014 [24] | PNG | Blood, urine, wounds | | | 43.9 (164) | | | | | | | | | | | | |
| ***Streptococcus pneumoniae*** | | | | | | | | | | | | | | | | | |
| 1968–1970 [30] | West Sepik | | 12 (530) | | | | | | | | | | | | | | |
| 1969 [28] | West Sepik | Throat swab | 100 (1) | | | | | | | | | | | | | | |
| 1971–1974 [28] | PNG | | 14 (292) | | | | | | | | | | | | | | |
| 1978 [31] | Port Moresby | | 33 (57) | | | | | | | 0 | 0 | 0 | | | | | |
| 1978–1981 [31] | Goroka | Blood, lung | 63 (24) | | | | | | | | | | | | | | |
| 1978–1987 [35] | Goroka | Blood, lung | 52 (73) | | | | | | | | | | | | | | |
| 1980–1982 [36] | Goroka | Nasal swabs | 63 (956) | | | | | | | | | | | | | | |
| 1980–1984 [33] | Goroka | CSF | 22 (67) | | | | | | | 0 | | | | | | | |
| 1980–1985 [17] | Goroka, Port Moresby | Multiple † | 56 * (3018) | | | | | | | 0 (1047) | | | | | | | |
| 1980–1987 [38] | Goroka | Nasal swabs, invasive isolates | 67 (898) | | | | | | | | | | | | | | |
| 1982–1983 [16] | Goroka | Skin | 60 (5) | | | | | | | 0 | | | | | | | |
| 1983–1984 [34] | Goroka | Blood | 46 (28) | | | | | | | 0 | | | | | | | |

| | | | | | | | | | | | | |
|---|---|---|---|---|---|---|---|---|---|---|---|---|
| 1985–1987 [35] | Goroka | Blood, lung | | | | | | 0 (655) | 0 (655) | 0 (655) | 0 (655) | |
| 1989–1992 [39] | Goroka | Blood, CSF | 23 (31) | 22 (46) | | | | 0 | | 0 | 74 (61) | 65 (32) |
| 1996–2005 [42] | Goroka | CSF | 21.5 (177) | | | 0.8 * (124) | | 2.3 (176) | 4.2 (96) | | 4 (176) | |
| 1998–2000 [4] | Goroka | | 100 (1) | | | | | 0 (1) | | | | |
| 2000 [41] | Goroka | | | | | | | 100 (2) | | | | |
| 2006–2009 [41] | Madang | Blood, CSF | 13.3 (15) | | | 0 (15) | | | 6.3 (16), 25 * (16) | | | 17.7 (17), 41 * (17) |
| 2009 [4] | Port Moresby | | 7 (40) | | 0 (13) | 0 (6) | 93.9 (33) | 8 (38) | 4 (28) | 0 (40) | 16.7 (6) | |
| ***Streptococcus pyogenes*** | | | | | | | | | | | | |
| 1983 [16] | Goroka | Skin | 0 (337) | | | | | | | | | |
| 1984–1986 [17] | Goroka | Multiple † | 0 | | | | | 0 | 0 | | | |
| ***Streptococcus agalactiae*** | | | | | | | | | | | | |
| 1982–1983 [16] | Goroka | Skin | 0 (2) | | | | | | | | | |
| **Other β-hemolytic streptococci** | | | | | | | | | | | | |
| 1984–1986 [17] | Goroka | Multiple † | 0 (22) | | | | | 5 (22) | 15 (20) | | | |
| ***Corynebacterium* spp.** | | | | | | | | | | | | |
| 1984–1986 [17] | Goroka | Skin | 0 (8) | | | | | 0 (8) | 0 (8) | 0 (8) | | |

PCN = penicillin, CLX = (flu)cloxacillin, MET = methicillin, OXA = oxacillin, AMP = ampicillin/amoxicillin, CTX = ceftriaxone, GEN = gentamicin, KAN = kanamycin, CMP = chloramphenicol, TET = tetracycline, ERY = erythromycin, COT = cotrimoxazole, TMP = trimethoprim–sulfamethoxazole, CLN = clindamycin, CIP = ciprofloxacin, RIF = rifampicin. * = intermediate resistance, † = blood, CSF, urine, skin lesions, stool, joint aspirates, lung aspirates.

**Table S2.** Antimicrobial resistance among Gram-negative organisms in Papua New Guinea.

| | | | % Resistance to Antibiotic (No. of Isolates Evaluated) | | | | | | | | | | | | | |
|---|---|---|---|---|---|---|---|---|---|---|---|---|---|---|---|---|
| Year | Location | Tissue | PCN | MET | AMP | CTX | GEN | KAN | STR | CMP | TET | ERY | COT | TMP | CIP | NAL | SPC |
| ***Haemophilus influenzae*** | | | | | | | | | | | | | | | | | |
| 1980–1984 [33] | Goroka | CSF | 1.8 (56) | | 0 | | | | | 0 | | | | | | | |
| 1981 [32,50] | Goroka | CSF, nasopharynx | 0.7 (293) | | | | | | | 0 (2) | | | | | | | |
| 1981–1986 [17] | Goroka | Multiple † | 0.3 (1516) | | 0.3 (1516) | | | | | | | | | | | | |
| 1983–1984 [34] | Goroka | Multiple † | 0 (30) | | 0 (30) | | | | | | | | | | | | |
| 1983–1986 [17] | Goroka | Multiple † | | | | | | | | 0 (839) | | | | | | | |
| 1989–1992 [39] | Goroka | Blood, CSF | | | 13 (24) | | | | | 0 | 0 | | 28 (25) | 44 (9) | | | |
| 1996–2005 [42] | Goroka | CSF | | | 28.4 (162) | 5 (80) | | | | 31.5 (162) | | | 34 (162) | | | | |
| 1998 [51] | Lae, Goroka, Port Moresby | | | | | | | | | 25 | | | | | | | |
| 2000 [37] | Lae, Goroka, Port Moresby | | | | | | | | | 21 | | | | | | | |
| 2000 [20] | Goroka | | | | | | | | | 22.7 | | | | | | | |
| 2000 [4] | Goroka | | | | | | | | | 36.4 (11) | | | | | | | |

| | | | | | | | | | | | | | | |
|---|---|---|---|---|---|---|---|---|---|---|---|---|---|---|
| 2006–2009 [41] | Madang | Blood, CSF | 93.3 (15) | | | 0 (10) | | | | 100 (14) | 93.3 (15) | | 100 (15) | |
| ***Escherichia coli*** | | | | | | | | | | | | | | |
| 1978 [15] | Rabaul | Sputum | 100 (1) | 100 (1) | | | 0 (1) | | | 0 (1) | | | | |
| 1984–1986 [17] | Goroka | Multiple † | | | 46 (37) | | 3 (37) | 8 (13) | | 32 (27) | 11 (27) | 8 (37) | | |
| 1997–1998 [54] | Goroka | | | | | | 38 (8) | | | 88 (8) | | | | |
| 2012 [24] | PNG | Blood, stool, urine, pus | | | | 24.1 ‡ (174) | | | | | | | | 13.3 § (526) |
| ***Klebsiella* spp.** | | | | | | | | | | | | | | |
| 1978 [15] | Rabaul | Sputum | 100 (1) | 100 (1) | | | 0 (1) | | | 0 (1) | | | | |
| 1992 [56] | Port Moresby | Blood | 100 (1) | | 100 (1) | | 100 (1) | | 100 (1) | 100 (1) | 0 (1) | 100 (1) | | |
| 1992 [56] | Port Moresby | Blood | | | | | | | | 54 (28) | 29 (28) | 61 (28) | | |
| 1997–1998 [54] | Goroka | Blood, CSF, lung | | | | | 76 (14) | | | 100 (14) | | | | |
| 2007–2008 [57] | Port Moresby | Blood | | | | 54 ‡ (57) | | | | | | | | |
| 2008–2009 [21] | Madang | Blood | | | 100 (2) | | 50 (2) | | | 100 (2) | 100 (2) | 100 (2) | | 0 (2) |
| 2012 [24] | PNG | Blood, urine, stool, wounds, pus | | | | 63.5 ‡ (252) | | | | | | | | |
| ***Klebsiella* spp. and *Enterobacter* spp.** | | | | | | | | | | | | | | |
| 1984–1986 [17] | Goroka | Multiple † | | | 95 (22) | | 5 (22) | 6 (17) | | 45 (22) | 36 (11) | 32 (22) | | |
| ***Enterobacter* spp.** | | | | | | | | | | | | | | |
| 1997–1998 [54] | Goroka | | | | | | 57 (7) | | | 100 (7) | | | | |
| ***Proteus mirabilis*** | | | | | | | | | | | | | | |
| 1997–1998 [54] | Goroka | | | | | | 33 (3) | | | 100 (3) | | | | |
| ***Proteus* spp. and *Providencia* spp.** | | | | | | | | | | | | | | |
| 1984–1986 [17] | Goroka | Multiple † | | | 45 (11) | | 18 (11) | 33 (9) | | 82 (11) | 100 (10) | 55 (11) | | |
| ***Providencia* spp.** | | | | | | | | | | | | | | |
| 1997–1998 [54] | Goroka | | | | | | 0 (1) | | | 0 (1) | | | | |
| ***Morganella morganii*** | | | | | | | | | | | | | | |
| 1997–1998 [54] | Goroka | | | | | | 0 (2) | | | 0 (2) | | | | |
| ***Pseudomonas* spp.** | | | | | | | | | | | | | | |
| 1984–1986 [17] | Goroka | Multiple † | | | 55 (11) | | 0 (12) | 30 (10) | | 83 (12) | 50 (8) | 67 (12) | | |
| 1997–1998 [54] | Goroka | Blood, urine, stool, wounds, pus | | | | | 82 (11) | | | 100 (11) | | | | |
| 2008–2009 [21] | Madang | Blood | | | 100 (1) | | 0 (1) | | | 100 (1) | 100 (1) | 100 (1) | | 0 (1) |
| ***Acinetobacter* spp.** | | | | | | | | | | | | | | |
| 1997–1998 [54] | Goroka | | | | | | 0 (1) | | | 100 (1) | | | | |
| ***Burkholderia cepacia*** | | | | | | | | | | | | | | |
| 1997–1998 [54] | Goroka | | | | | | 100 | | | 67 (3) | | | | |
| ***Burkholderia pseudomallei*** | | | | | | | | | | | | | | |
| 1995–2005 [67] | Western Province | Clinical, environmental | | | 0 (39) | | | | | 48.7 (39) | 0 (39) | | | |
| ***Aeromonas* spp.** | | | | | | | | | | | | | | |
| 1984–1986 [17] | Goroka | Stool, skin | | | 75 (8) | | 0 (8) | | | 13 (8) | 0 (5) | 0 (8) | | |

| | | | | | | | | | | | | | | | | |
|---|---|---|---|---|---|---|---|---|---|---|---|---|---|---|---|---|
| 1997–1998 [54] | Goroka | | | | | 0 (1) | | | 100 (1) | | | | | | | |
| ***Citrobacter freundii*** | | | | | | | | | | | | | | | | |
| 1997–1998 [54] | Goroka | Blood, urine, stool, wounds, pus | | | | 100 (3) | | | 67 (3) | | | | | | | |
| ***Alcaligenes* spp.** | | | | | | | | | | | | | | | | |
| 1997–1998 [54] | Goroka | | | | | 0 (1) | | | 100 (1) | | | | | | | |
| 2008–2009 [21] | Madang | Blood | | 100 (1) | | 100 * (1) | | | 0 (1) | 100 (1) | | 0 (1) | | 100 * (1) | | |
| ***Shigella* spp.** | | | | | | | | | | | | | | | | |
| 1962–1963 [72] | Port Moresby | Stool | | | | | | 12.9 (70) | | 8.6 (70) | | | | | | |
| 1984–1986 [17] | Goroka | Stool | | 86 (94) | | 0 (94) | 0 (94) | | 83 (94) | 91 (54) | | 1 (94) | | | | |
| 2000–2009 [73] | Port Moresby | Stool | | 96 (98) | | | | | 60 (114), 27 * (114) | | | 86 (76) | | | 15 (13) | |
| 2010–2011 [74] | Goroka | Stool | | 91.5 (47) | 0 (47) | | | | 55.3 (47) | 76.6 (47) | | | 70.2 (47) | 0 (47) | | |
| 2014 [24] | PNG | Stool | | | | | | | | | | | | 0 § (53) | | |
| 2018 [75] | Oceania (60 samples from PNG) | Stool | | 70.8 (72) | | | | | 49 (53) | 59.7 (72) | | | 54.2 (72) | | 6 (16) | |
| ***Salmonella* spp.** | | | | | | | | | | | | | | | | |
| 1984–1986 [17] | Goroka | Stool | | 53 (38) | | 0 (38) | 37 (38) | | 58 (38) | 6 (33) | | 3 (38) | | | | |
| 2010–2011 [74] | Goroka | | | 80 (5) | 0 (5) | | | | 40 (5) | 60 (5) | | | 60 (5) | 0 (5) | | |
| 2014 [24] | PNG | | | | | | | | | | | | | 33.3 § (15) | | |
| ***Campylobacter* spp.** | | | | | | | | | | | | | | | | |
| 1984–1986 [17] | | | | 24 (55) | | 0 (55) | | | 0 (55) | 0 (55) | | 100 (55) | | | | |
| ***Vibrio cholerae*** | | | | | | | | | | | | | | | | |
| 2009 [79] | PNG | Stool | | | | | | | | 27.8 * (36) | | | | | | |
| 2009–2011 [79] | PNG | Stool, rectal swabs | | 75.8 (302), 17.2 * (302) | | | | | 3.1 (255), 1.6 * (244) | | | 3.2 (282), 1.4 * (282) | | 1 (305), 0.7 * (305) | 0.3 (300) | |
| 2010 [79] | PNG | Stool | | | | | | | | 50.5 * (212) | | | | | | |
| 2011 [79] | PNG | Stool | | | | | | | | 11.8 * (51) | | | | | | |
| 2011 [79] | PNG | Stool | | | | | | | | 9.7 (299) | 38.2 (254) | | | | | |
| ***Neisseria meningitidis*** | | | | | | | | | | | | | | | | |
| 1984–1986 [17] | Goroka | Blood, CSF | 0 (5) | | | | | | 0 (5) | | | | | | | |
| 2009 [44] | Port Moresby | | 0 | | 0 | | | | | 33 (3) | | | | | | |
| ***Neisseria gonorrhoeae*** | | | | | | | | | | | | | | | | |
| 1989–1990 [83] | Port Moresby, Goroka, Rabaul, Lae, Daru | | 44 | | | | | | | | | | | | | |
| 1993 [85] | PNG | | 12.5 (40) | | | | | | | 7.5 (40) | | | | 0 § (40) | | 3.3 (30) |
| 1994 [84] | PNG | | 8.7 (218) | | | | | | | 4.1 (218) | | | | 5 § (218) | | 1.8 (57) |

| | | | | | | | | | |
|---|---|---|---|---|---|---|---|---|---|
| 2004–2005 [87] | Port Moresby, Goroka, Lae, Mt Hagen | 40 (52) | 0 (52) | 0 (52) | 19 (52) | 0 (52) | 2 (52) | | 0 (52) |
| 2005 [86] | Port Moresby | 61.1 | | 0.7 | 49 | | 1.2 | 5 | 0.7 |
| 2006 [86] | Port Moresby | 64.7 | | 0 | 17.7 | | 1.5 | 2.9 | 0 |

PCN = penicillin, MET = methicillin, AMP = ampicillin/amoxicillin, CTX = ceftriaxone, GEN = gentamicin, KAN = kanamycin, STR = streptomycin, CMP = chloramphenicol, TET = tetracycline, ERY = erythromycin, COT = cotrimoxazole, TMP = trimethoprim–sulfamethoxazole, CIP = ciprofloxacin, NAL = nalidixic acid, SPC = spectinomycin, * intermediate resistance, † blood, CSF, urine, skin lesions, stool, joint aspirates, lung aspirates, ‡ unspecified third-generation cephalosporin resistance, § unspecified fluoroquinolone resistance.
